# Axin1‘s mystique in manipulating microbiome amidst colitis

**DOI:** 10.1080/19490976.2023.2286674

**Published:** 2023-11-27

**Authors:** Shari Garrett, Monica C. Asada, Jun Sun

**Affiliations:** a Division of Gastroenterology and Hepatology, Department of Medicine, University of Illinois Chicago, Chicago, IL, USA; b Department of Microbiology and Immunology, University of Illinois Chicago, Chicago, IL, USA; c UIC Cancer Center, University of Illinois Chicago, Chicago, IL, USA; d Medicine, Jesse Brown VA Medical Center, Chicago, IL, USA

**Keywords:** Axin1, Axin2, beta-catenin, colitis, fecal microbiota transplantation, inflammatory bowel disease, Paneth cells, Goblet cells, microbiome, Wnt

## Abstract

Classically, Axin1 is considered a regulator of Wnt/β-catenin signaling. However, Axin1’s roles in host-microbial interactions have been unknown. Our recent study has demonstrated that deletion of intestinal epithelial Axin1 in epithelial cells and Paneth cells protects the host against colitis by enhancing *Akkermansia muciniphila*. Loss of intestinal epithelial or Paneth cell Axin1 results in increased Wnt/β-catenin signaling, proliferation, and cell migration. This is associated with morphologically altered goblet and Paneth cells, including increased Muc2 and decreased lysozyme. Axin1 deletion specifically enriched *Akkermansia muciniphila*. *Akkermansia muciniphila* in Axin1 knockout mice is the driver of protection against DSS-induced inflammation. Here, we feature several significant conceptual changes, such as differences between Axin1 and Axin2, Axin1 in innate immunity and microbial homeostasis, and Axin1 reduction of *Akkermansia muciniphila*. We discuss an important trend in the field related to Paneth cells and tissue-specific Axin1 manipulation of microbiome in health and inflammation.

## Introduction

Axin1 is known as a negative regulator of the canonical Wnt/β-catenin pathway. It forms a complex with adenomatous polyposis coli (APC), glycogen synthase kinase-3 (GSK3), and casein kinase-1 (CK1). The complex phosphorylates β-catenin, triggering ubiquitination and ultimately leads to the degradation of β-catenin. The Wnt/β-catenin pathway contributes to a wide range of cellular activities, including embryonic development, cell proliferation, tissue regeneration, stem cell maintenance, and cell fate determination. Dysregulation of the Wnt/β-catenin pathway due to Axin1 mutations is associated with various cancers, especially gastrointestinal (GI) cancers, such as hepatocellular carcinoma (HCC), gastric cancer, and colorectal cancer. For example, Axin1 alteration is found in 8–10% of HCCs.^1,2^ However, little is known regarding its specific function in host-microbial interactions. In a recent study published in *Engineering*^3^, Garrett et al. have demonstrated a new role for intestinal epithelial Axin1 in colitis by reducing *Akkermansia muciniphila (A. muciniphila)*, thus making the host susceptible to colitis. Conditional deletion of Axin1 in epithelial cells and Paneth cells protects against colitis with enriched *A. muciniphila* in the microbiome community. In the current commentary, we will highlight several significant conceptual changes related to the role of Axin1 in innate immunity and microbial homeostasis.

## Axin1 contributes to intestinal inflammation by regulating the microbiome, not just through the Wnt/β-catenin pathway

When the Wnt/β-catenin pathway is activated, the Axin1-centered complex will be sequestered (note that it’s not destroyed). As a result, free β-catenin in the cytosol can translocate into the nucleus and initiate transcription by interacting with transcription factors, TCF/LEF; as a result, one of the endogenous Wnt target genes, as well as an Axin1 homolog, Axin2, is induced following pathway activation. Sanson *et al*.^4^ suggest that the function of Axin1 in the Wnt/β-catenin pathway can be entirely compensated for by Axin2, and vice versa. It remains a subject of debate as other studies describe otherwise. Thorvaldsen *et al*. have observed that in tankyrase (TNKS) inhibitor-induced degradation of β-catenin, Axin2 plays a more significant part in forming degradosome in CRC cell lines, indicating that Axin2 might have a greater role in turning the pathway off while Axin1 is more dominant in the turn-on process.^5^ Roo *et al*. also emphasize that Axin2 is crucial and non-redundant for precise hematopoiesis and T lymphopoiesis, as demonstrated in a mTurquoise2-reporter mouse model, suggesting its fine-tuning function is irreplaceable by Axin1.^6^ Overall, Axin1 may appear genetically redundant with Axin2 in the Wnt/β-catenin pathway, as they can act rather dissimilarly on proteostatic and transcriptional levels, and their effects can also be highly tissue-specific.^6^ For instance, Axin2 knockout mice show dental and cranial agenesis, whereas Axin1 knockout has proved to be lethal.^7–9^ Previous studies *in vitro* have demonstrated that Axin2 is not involved in inhibiting intestinal cell bacterial invasion and inflammation^10^. It is unknown how Axin2 may contribute to intestinal homeostasis, microbiome, and inflammation *in vivo*.

Axin1 might be more than a mere gatekeeper in the Wnt/β-catenin pathway.^11^ A study utilizing RNA-seq has unveiled that Axin1 is linked to IFN-γ/STAT1/Th1 immune program through an unidentified mechanism; moreover, CRC patients exhibiting an Axin1-dependent gene expression signature tend to have a more favorable prognosis.^4^ Guo *et al*. propose that Axin1 enhances the antiviral response to influenza by activating the JNK/c-Jun and Smad3 pathways, reducing virus replication, and increasing interferon (IFN) production. Overexpressing Axin1 or using the Axin1 stabilizer XAV939 has been shown to decrease influenza virus replication in lung epithelial cells.^12^ Our previous study has demonstrated that pathogenic *Salmonella* manipulates Axin1 signaling for its infection and invasion *in vitro*^13^. In our current paper^3^, we found that Axin1 knockout alleviates DSS-induced colitis via alteration of intestinal environment and microbiota. These findings imply that Axin1 could play multiple roles in different signaling pathways, such as pro-inflammation and anti-tumor responses.

In the Axin1 intestinal epithelial cell (IEC) knockout *Axin1^Δ^*^IEC^ mice, we found increased intestinal epithelial Axin2, the Wnt target gene, at the mRNA level. The absence of intestinal epithelial Axin1 altered Wnt/β-catenin signaling and enhanced proliferation, which may contribute to protection from DSS injury as well. This cellular protection is likely to be microbiome-dependent because co-housing *Axin1^Δ^*^IEC^ and *Axin1*^LoxP^ mice reduced *A. muciniphila* in *Axin1^Δ^*^IEC^ mice and made them more susceptible to colitis. Moreover, *A. muciniphila* treatment protected against DSS-induced colitis in *Axin1*^LoxP^ mice. Thus, intestinal epithelial Axin1 contributes to inflammation by regulating the microbiome, not just through the Wnt/β-catenin pathway.

## New findings of Axin1 in inflammatory bowel disease (IBD)

There is limited study on Axin1 links to human IBD. Our paper reported increased Axin1 expression at both the mRNA and protein levels in human IBD, including ulcerative colitis (UC), and Crohn’s disease (CD). Specifically, we identified increased Axin1 expression positively correlated with pro-inflammatory cytokines IL-6 and TNF-α in human CD. Dysfunction of Wnt/β-catenin was reported in human IBD and Axin1 serum level was elevated in patients with UC^14^. However, the focus of previous studies is on the host impacts mediated through the Wnt/β-catenin pathway. For the first time, our study identifies links between the gut microbiota and intestinal Axin1 in intestinal inflammation through tissue-specific deletion of Axin1 in intestinal epithelium and Paneth cells. Loss of intestinal Axin1 plays a novel role in intestinal inflammation by altering Paneth cells and microbiome (e.g., enriched *A. muciniphila)*. Investigation of the interactions between microbiota and Axin1 will provide novel mechanistic strategies for therapeutic approaches for human IBD.

## Axin1 deletion leads to altered Paneth cells

Loss of intestinal epithelial or Paneth cell (PC) Axin1 results in increased Wnt/β-catenin signaling, proliferation, and cell migration. This is associated with altered goblet and Paneth cell morphology with increased MUC2 and decreased lysozyme, respectively. PCs can regulate innate immunity by sensing the microbiota and adjusting their production of antimicrobial peptides, like lysozyme^15,16^. Axin1 is a known regulator of Wnt/β-catenin signaling which promotes the differentiation and maturation of PCs, thereby regulating antimicrobial peptide production. Specifically, mice lacking the Wnt/β-catenin signaling regulator TCF-1 demonstrated a decrease in lysozyme^17^. In addition, mice lacking PC lysozyme have increased goblet cells and Muc2 expression^18^. Axin1 may regulate lysozyme expression in a Wnt/β-catenin dependent fashion. Perturbations of PC lysozyme function in the lyz1^−/−^ mice increase mucolytic bacteria, e.g., *Ruminococcus gnavus*, alter the gut community landscape, and protect against DSS-induced colitis^18^. In addition, increased lysozyme has been observed in patients with UC^19,20^. We have demonstrated that PC Axin1 maintains intestinal and microbial homeostasis, which may be the driving factor in protection against colitis. Axin1 deficiency leads to alterations in the gut microbiome (e.g., enriched *A. muciniphila*) due to decreased lysozyme processing of the gut microbiota and a potential anti-colitogenic type 2 immune response as seen in Lyz1^−/−^ mice^18^. Further *ex vivo* and *in vitro* studies may narrow down the cellular role of Axin1 in PC function.

The tissue-specific role of Axin1 in immunity is still unknown. A recent study reported that Axin1 stimulates intestinal inflammation via an interferon-gamma/Th1 program that prevents tumor growth^4^. Our paper has demonstrated that Axin1 and the microbiome are promising targets for colitis. Axin1 status in the epithelium is critical for PC function, but how Axin1 regulates the immune system in IBD is not fully understood. It has been shown that mice with deficiency in LRP5/6 in CD11c+ cells, that have a stabilized form of β-catenin, decreased DSS-induced colitis severity, and reduced inflammatory cytokines^21^. Many other studies support the anti-inflammatory role of β-catenin in other mouse models. Specifically, Wnt signaling in dendritic cells can regulate intestinal immunity by promoting T-cell responses and secretion of cytokines to a tolerogenic state^22^. It is unknown whether Axin1 plays a similar role in CD11 myeloid cells in the context of colitis. Besides Paneth and intestinal epithelial cells, further study is required to determine whether myeloid Axin1 plays a role in intestinal homeostasis and inflammation. Additional *in vivo* studies would also determine an overall immune profile in Axin1-deficient mice. Furthermore, we have developed a myeloid cell Axin1 knockout mouse model. Explorations in myeloid Axin1 may elucidate its mechanistic role in modulating- gut microbiota.

## Axin1 regulation of mucus barrier and *Akkermansia muciniphila*

Axin1-specific alterations in intestinal mucosa and thinning of the mucus barrier lead to enriched *A. muciniphila* and goblet cells. The presence of *A. muciniphila* in Axin1 knockout mice is the driver of protection against DSS-induced inflammation. In addition, the loss of *Muribaculaceae* during co-housing is another contributing factor to DSS colitis susceptibility.

**Figure 1.**
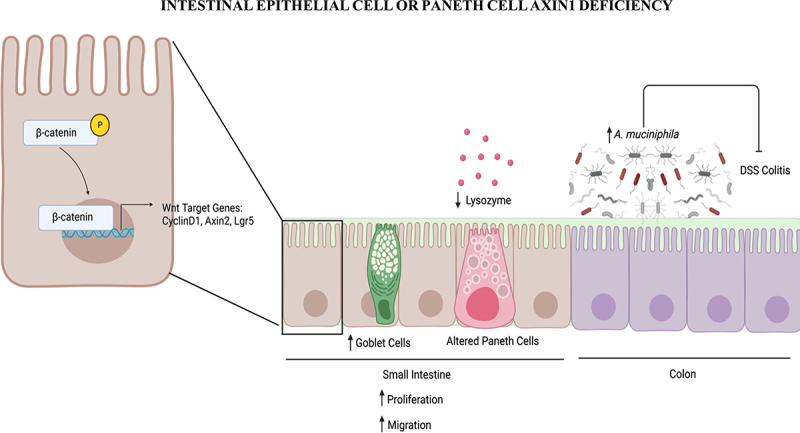
Intestinal epithelial Axin1 protection against colitis via gut microbiota alterations.

We found that *Axin1^Δ^*^IEC^ mice are protected from DSS-induced inflammation with increased MUC2. We found increased MUC2 and goblet cells in *Axin1^Δ^*^IEC^ mice. Axin1 knockout in IECs did not significantly change intestinal permeability (data not shown). Moreover, *A. muciniphila* was enhanced in *Axin1^Δ^*^IEC^ mice. MUC2 likely provides a favorable environment to stimulate the abundance of *A. muciniphila*. Thus, increased MUC2 and *A. muciniphila* collectively may have granted the *Axin1^Δ^*^IEC^ mice increased colitis protection compared with controls (Figure 1). It has been widely reported that *A. muciniphila* and *Muribaculaceae* are underrepresented in metabolic and neurodegenerative disorders and intestinal inflammation^23–26^. There are trends to explore these strains as new probiotics for disease prevention and treatment.

## Future directions

Garrett *et al*. demonstrate a novel and critical role of Axin1 in regulating intestinal epithelial development and microbial homeostasis^3^. Our study has provided insights into the molecular mechanism that might contribute to IBD. There are no human studies assessing the role of intestinal epithelial and Paneth cell Axin1 in inflammation and microbiome. It is unknown how Axin1 may directly regulate PC function to protect against DSS injury. Increased Wnt/β-catenin signaling and intestinal proliferation may be a contributing factor, and further investigations in the colon may elucidate this mechanism. Moreover, dissecting the colonic mechanisms of Axin1 may elucidate its role in intestinal homeostasis and colorectal cancer^4,27,28^. Microbiome and spatially resolved metabolomics analysis reveal the anticancer role of intestinal *A. muciniphila* by crosstalk with intratumoral microbiota and reprogramming tumoral metabolism in a lung cancer model^29^. Explorations in the role of myeloid Axin1 can potentially expound the tissue-specific function of Axin1 in auto-immune diseases. Overall, further investigation of the microbiota-Axin1 interactions will provide novel insights into the development of chronic diseases, e.g., IBD and colon cancer, and mechanistic strategies for therapeutic approaches.

Loss of intestinal epithelial or Paneth cell Axin1 results in increased Wnt/β-catenin signaling, proliferation, and cell migration. This is associated with altered goblet and Paneth cell morphology with increased MUC2 and decreased lysozyme, respectively. Axin1-specific deletion in the intestinal mucosa leads to an altered gut microbiome (e.g., enriched *A. muciniphila)* and thinning of the mucus barrier. The presence of *A. muciniphila* in Axin1 knockout mice is the driver of protection against inflammation.
